# How to Explore an Endocrine Cause of Hypertension

**DOI:** 10.3390/jcm11020420

**Published:** 2022-01-14

**Authors:** Jean-Baptiste de Freminville, Laurence Amar

**Affiliations:** 1 Paris-Cardiovascular Research Center, INSERM, UMR970, Université de Paris, F-75006 Paris, France; 2 Unité Fonctionnelle D’HYPERTENSION Artérielle, Centre de Référence des Maladies Rares de la Surrénale, Hôpital Européen Georges Pompidou, Assistance Publique-Hôpitaux de Paris, F-75015 Paris, France

**Keywords:** hypertension, aldosteronism, pheochromocytoma, secondary hypertension

## Abstract

Hypertension (HTN) is the most frequent modifiable risk factor in the world, affecting almost 30 to 40% of the adult population in the world. Among hypertensive patients, 10 to 15% have so-called “secondary” HTN, which means HTN due to an identified cause. The most frequent secondary causes of HTN are renal arteries abnormalities (renovascular HTN), kidney disease, and endocrine HTN, which are primarily due to adrenal causes. Knowing how to detect and explore endocrine causes of hypertension is particularly interesting because some causes have a cure or a specific treatment available. Moreover, the delayed diagnosis of secondary HTN is a major cause of uncontrolled blood pressure. Therefore, screening and exploration of patients at risk for secondary HTN should be a serious concern for every physician seeing patients with HTN. Regarding endocrine causes of HTN, the most frequent is primary aldosteronism (PA), which also is the most frequent cause of secondary HTN and could represent 10% of all HTN patients. Cushing syndrome and pheochromocytoma and paraganglioma (PPGL) are rarer (less than 0.5% of patients). In this review, among endocrine causes of HTN, we will mainly discuss explorations for PA and PPGL.

## 1. Introduction

Hypertension (HTN) is the most frequent modifiable risk factor in the world, affecting almost 30 to 40% of the adult population in the world. In 2015, 1.13 billion persons had HTN. This proportion increases with age [1]. In 2017, it was accountable for 10.4 million annual deaths and 218 million disability-adjusted life years (DALYs) [2].

Among hypertensive patients, 10 to 15% have so-called “secondary” HTN, which means HTN due to an identified cause. The most frequent secondary causes of HTN are renal arteries abnormalities (renovascular HTN), kidney disease, and endocrine HTN, which are primarily due to adrenal causes.

Knowing how to detect and explore endocrine causes of hypertension is particularly interesting because some causes have a cure or a specific treatment available. Moreover, the delayed diagnosis of secondary HTN is a major cause of uncontrolled blood pressure [3]. Therefore, screening and exploration of patients at risk for secondary HTN should be a serious concern for every physician seeing patients with HTN.

Regarding endocrine causes of HTN, the most frequent is primary aldosteronism (PA), which also is the most frequent cause of secondary HTN and could represent 10% of all HTN patients. Cushing syndrome and pheochromocytoma and paraganglioma (PPGL) are rarer (less than 0.5% of patients).

In this review, among endocrine causes of HTN, we will mainly discuss explorations for PA and PPGL.

## 2. Primary Aldosteronism and Other Forms of Mineralocorticoid Excess

### 2.1. Primary Aldosteronism

#### 2.1.1. Definition and Epidemiology

Primary aldosteronism (PA) is defined as an inappropriate secretion of aldosterone that is autonomous from sodium intake, potassium levels, and renin-angiotensin activity. The consequences of this autonomous secretion result in sodium and water retention, hypertension (HTN), increase in urinary potassium excretion, and sometimes hypokalemia.

PA is the leading cause of secondary HTN. Its prevalence is estimated around 10% of patients with HTN. This prevalence varies among studies depending on diagnosis criteria and on the population of the patients included: in experts and specialized centers where most of the studies were conducted, the proportion varies from 10 to 18% [4], whereas in a population of patients treated by general practitioners, the prevalence was 5.9% [5]. This proportion increases with the severity of HTN and approaches 20% in patients with resistant HTN [6]. Moreover, the number of patients is very likely to be underestimated because diagnosis is complex. Moreover, there is a continuum between low-renin primary HTN and PA, and the varying cut-offs among the studies result in subjective interpretations.

#### 2.1.2. Who Should Be Screened?

According to French and European guidelines [7,8], screening for PA should be based on clinical biological and radiological characteristics. Practically, screening should be performed in the following situations:

- Resistant HTN, meaning systolic blood pressure (SBP) ≥ 140 mmHg or diastolic blood pressure (DBP) ≥ 90 mmHg despite at least three medications including thiazides, renin-angiotensin system blockers (RASb), and calcium channel blockers (CCB);
- Grade 3 HTN, meaning SBP ≥ 180 mmHg or DBP ≥ 110 mmHg [6];
- Grade 2 HTN, especially if there is a poor treatment response, as prevalence increases with HTN severity [6];
- HTN at young age (beginning < 40 years old);
- Hypokalemia, whether it is associated or not with diuretic intakes;
- Adrenal incidentaloma;
- Family history of PA (although monogenic forms are rare);
- Atrial fibrillation unexplained by structural heart disease and other conditions such as hyperthyroidism;
- Early stroke or disproportionate renal or cardiovascular (CV) complications regarding age or the severity of HTN. Indeed, patients with PA have higher CV and renal morbidity.

It is worthy to note that the absence of hypokalemia does not allow ruling out PA. Indeed, around 60% of patients do not have hypokalemia [4].

Likewise, the absence of adrenal mass does not rule out PA as up to 50% of patients display normal adrenals [9].

Moreover, recent recommendations [8] take into account the fact that the development of PA is probably gradual and that patients with mild phenotypes may develop a more severe disease later, with a more marked phenotype (hypokalemia). However, the impact of not screening these patients on CV mortality is likely but not known.

Regarding young age, PA is not more frequent in younger patients and the median age of diagnosis is around 50 years [5], but cardiovascular benefits from treatment might be more important in young patients. However, the age cut-off still needs to be adjusted.

A new statement concerns atrial fibrillation. Until most recent recommendations, atrial fibrillation was not an indication to screen patients for PA. However, the PAPHHY study showed that PA is highly prevalent in hypertensive patients with atrial fibrillation with no other identifiable cause of arrhythmia [10].

#### 2.1.3. How to Diagnose Primary Aldosteronism?

Diagnostic strategy for PA is complex because of the multiple dosage methods of renin and aldosterone and because units and norms vary among laboratories.

The diagnostic approach recommended by the Endocrine Society guideline requires two steps: screening followed confirmatory/exclusion testing. If diagnosis is confirmed, a third step can be the subtype diagnosis to distinguish unilateral from bilateral disease.

##### Screening

Screening for PA is based on the detection of a dissociation between aldosterone and renin secretion by measuring the aldosterone-to-renin ratio (ARR).

Measurements of plasma renin are mostly direct renin concentration (DRC) by radioimmunoassay and sometimes by chemiluminescence. Rare centers still use plasma renin activity (PRA), expressed in nanograms per milliliters per minute (ng/mL/min) or picomoles per liter per minute (pmol/L/min), but this measure is almost abandoned. The results for DRC are expressed in milli unit interval per liter (mUI/L). It is important to observe that both results can be flawed with low temperature (cryoactivation of plasma prorenin in renin); therefore, samples should be collected and processed at room temperature [11].

Plasma aldosterone can not only be measured by radioimmunoassay and immunometric techniques but also more recently by ultra-high liquid chromatography and tandem mass spectrometry in plasma and 24-hour urine samples [12]. This technique avoids crossed reaction with other mineralocorticoids and is, therefore, more specific.

Renin and aldosterone measurements require standardized conditions because many of factors can interfere with the renin-angiotensin system (RAS) and, therefore, modify both renin and aldosterone concentrations. The recommendations for renin and aldosterone measurements are as follows [13]:

1. During morning fasting;
2. In a sitting position for at least 15 min;
3. Normal sodium diet (urinary sodium between 100 and 200 mmol/24 H if available);
4. Normal kalemia;
5. Interruption of interfering medications [14] (Table 1):
  - Diuretic, betablockers, angiotensin-converting enzyme inhibitors (ACEi), and angiotensin2 receptor blockers (ARB) should be stopped 15 days before;
  - Mineralocorticoid receptor antagonists (MRA) (spironolactone and eplerenone) should be stopped 45 days before;
  - Estroprogestative contraception should be stopped but also are not recommended in hypertensive patients;
  - Nonsteroidal anti-inflammatory should be stopped 15 days before but also are not recommended in hypertensive patients.

If necessary, antihypertensive medications with minimal impact on ARR can be prescribed (non-dihydropyridine calcium channel blockers, alpha-blockers and centrally acting agents). It also can be necessary to provide high dosages of potassium supplementation to avoid hypokalemia which, in addition to the life-threatening risk of arrhythmia, inhibits aldosterone secretion.

ARR is the parameter with the best sensitivity (68–94%) for screening PA compared to other biological markers taken separately [15].

Due to the pulsatile secretion of aldosterone, the dosages must be repeated [16]. At least two measurements must be performed in patients with low renin profile.

The interpretation of the ARR is complex due to the heterogeneity of dosage methods for measuring renin and aldosterone. Cutoffs vary depending on the dosage method. Moreover, because of the lack of accuracy of DRC measurements when it is low and to avoid ARR elevation only due to very low renin, a minimum value for DRC is set to 5 mUI/l [12].

There is no international consensus for ARR cut-offs.

The conversion factors are as follows:

1. Aldosterone: 1 pg/mL = 2.77 pmol/L;
2. Renin: 1 pg/mL = 1.67 mUI/L.

When aldosterone is measured by mass spectrometry, studies suggest that the ARR cutoff for PA diagnosis would decrease 30% [12,17]. Therefore, in our center, the cutoff is 46 pmol/mUI [12].

##### Diagnosis Confirmation/Exclusion

ARR has good sensibility on the diagnosis of PA but low specificity (some studies suggest that only one-third of patients with an elevated ARR have PA [18].

Therefore, a confirmatory test can be necessary.

According to the recommendations of the European Society of Hypertension [8], as the specificity for PA diagnosis increases with ARR value [19], a patient with spontaneous hypokalemia, plasma aldosterone greater than 20 ng/dL (550 pmol/L), and renin (PRA or DRC) below detection limit is considered to possess PA without needing a confirmatory test.

French and European recommendations, however, which we use in our center, proposed the following [20]:

1. PA is confirmed if ARR is above the laboratory cutoff twice in a row, with at least one dosage of plasma aldosterone greater than the cut-off;
2. PA can be ruled out if aldosterone is lower than 240 pmol/L (radioimmunoassay) or 157 pmol/L (mass spectrometry).

In other cases (when PA is suspected, but cannot be affirmed), a confirmatory test should be performed.

The goal of a suppressive dynamic test is to demonstrate the non-suppressibility of aldosterone production. Many tests exist, such as the saline infusion test, oral sodium loading test, fludrocortisone suppression test, or captopril challenge test [21]. There is no sufficient evidence to recommend one test over another. However, the seated sodium infusion test, which consists in infusion 2 L of 0.9% saline solution in 4 h and comparing aldosterone before and after infusion (diagnosis is confirmed if aldosterone is greater than 230 pmol/L in radioimmunoassay or 150 pmol/L in mass spectrometry), appears to be reliable and less complicated than other tests [20,22,23]. In the case of patients with renal insufficiency or heart failure and those at risk of fluid overload, the captopril challenge test is a good alternative.

##### Subtype Diagnosis

Once PA diagnosis is confirmed, it can be appropriate to investigate the subtype. The main subtypes of PA are unilateral forms (which can be accessible to surgery) and bilateral forms (only accessible to medical therapy).

**Figure 1:**
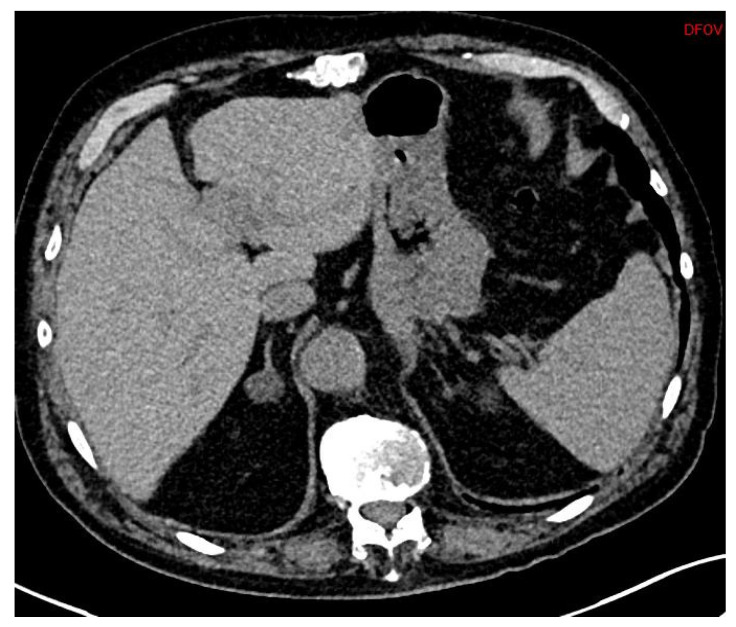
Abdominal CT (DFOV 80 × 35 mm): Adrenal adenoma, right adrenal nodule.

First, adrenal imaging is required, and the preferred method is computed tomography (CT) scanning with contrast, because the spatial resolution is better than magnetic resonance imaging (MRI)’s. CT scanning detects adrenal adenomas (Figure 1) but provides no information about aldosterone secretion and, therefore, cannot distinguish between aldosterone-producing adenomas (APA) and non-secretory adrenal adenomas. Moreover, micro-APAs are frequent in patients with confirmed PA and can be missed by adrenal imaging. It is, however, useful to exclude malignant adrenal tumors such as carcinoma, which might in rare cases produce aldosterone [24].

APAs are classically unilateral adrenal tumors from 10 to 20 mm, with a density of less than 10 UH. However, unilateral forms of PA can also occur as unilateral hyperplasia or even normal adrenal glands. A study of 950 patients showed that imaging techniques (CT or MR) were discordant compared with adrenal vein sampling (AVS) in 38% of cases (15% of cases were classified as unilateral instead of bilateral, 19% of cases were incorrectly classified as bilateral when they were unilateral, and even 4% of cases were classified as unilateral but on the wrong side) [25].

In conclusion, imaging alone is not sufficient to diagnose PA subtypes.

##### Adrenal Vein Sampling

Adrenal vein sampling (AVS) is the gold standard exam to affirm unilateral secretion of aldosterone.

This exam is realized under local anesthesia and by inserting a catheter by the femoral vein to the left and right adrenal veins. Cortisol and aldosterone are sampled for the left and right adrenal vein and from the femoral vein. As for plasma renin and aldosterone measurement, interfering treatments should be stopped 2 to 4 weeks before the exam (6 weeks for MRA) [26].

The exam should be performed by an experienced team, and the patient informed of the possibility of failure, mostly due to the difficulty to catheterize the right adrenal vein, which drains directly in the inferior vena cava [27]. The rate of failure is around 10% in well-trained teams.

Criteria for interpretation of AVS are as diverse as criteria for interpreting ARR [25,28]. It is recommended to check the selectivity index to demonstrate correct cannulation of the adrenal veins, and then to check the lateralization index to affirm unilateral secretion.

The selectivity index is the ratio of cortisol in the adrenal veins on the cortisol of the femoral vein, and AVS is considered valid if selectivity index is higher than 2. Otherwise, the results of the AVS cannot be interpreted.

The lateralization index corresponds to the ratio of ratios aldosterone on cortisol between the dominant and other adrenal vein. There is no undisputable cutoff for this index, but most consensus consider that lateralization can be affirmed when the index is superior to 4 [25].

Complications of AVS are rare but must be explained to patients (adrenal hematoma, femoral hematoma, and complications due to contrast enhancement product) [26].

AVS is considered as the goal standard for subtyping PA. However, several studies have shown that the interpretation of AVS is different among clinical teams [29]. Moreover, a randomized study has evaluated AVS versus CT for subtyping PA. The main objective was BP control after one year. This study showed that AVS was not superior to CT; however, patients with lateralized and non-lateralized AVS were included in the study, and 20% of patients operated on the presence of an adrenal nodule without an AVS were not cured by surgery. The authors concluded that their findings challenge current recommendations to perform AVS in all patients with PA [30].

#### 2.1.4. Genetics

One to five percent of PA may be familial forms, and others are sporadic. However, familial forms should be evocated for young patients with PA (<20 years) or with patients with first degree relatives with PA. Four types of familial PA are known until now, from type 1 to type 4 [31,32,33].

##### Germline Mutations

Glucocorticoid remediable aldosteronism (GRA), also known as familial hyperaldosteronism type 1 (FH-I), is a result of the chimeric CYP11B1/CYP11B2 gene, which causes an ectopic expression of aldosterone synthase activity under the regulation of ACTH, resulting in hyperaldosteronism. The diagnosis is based on amplification of the chimeric gene in polymerase chain reaction (PCR). The treatment consists in a low dose of dexamethasone in order to suppress ACTH, which may be associated to mineralocorticoid receptor blockers [32,34].

FH-II is due to germline mutation in CLCN2, which encodes the chloride channel ClC2. FH-III is due to germline mutations in KCNJ5, which encode for a potassium channel GIRK4. Moreover, germline mutations in CACNA1H, which encode the pore-forming α1 subunit of the T type voltage dependent calcium channel Cav3.2, are responsible for FH IV.

##### Somatic Mutations

Regarding sporadic forms, somatic mutations in four genes have been identified in nearly 60% of sporadic APAs (KCNJ5, ATP1A1, ATP2B3, and CACNA1D) [34]. Studies published recently, which performed genetic testing on adrenal nodules expressing aldosterone synthase using immunohistochemistry guided next generation sequencing (NGS), show that somatic mutations in APA driver genes are found in up to 90% of patients [32].

#### 2.1.5. Treatment

The treatment of PA has three objectives:

1. Normalize aldosterone secretion;
2. Normalize potassium;
3. Optimize Blood pressure or cure HTN.

The objectives on potassium and blood pressure are easy to understand, given the risk of cardiovascular complications due to hypokalemia (atrial fibrillation, rhythm disorders) and hypertension (cardiovascular events, stroke, ischemic cardiomyopathy, chronic kidney disease, etc.).

Moreover, Aldosterone hypersecretion may be deleterious. Indeed, some studies showed that patients with PA, with similar blood pressure levels, had more cardiovascular complications than patients with primary hypertension [26,35,36].

##### Surgical Treatment

Surgical treatment is recommended for patients with unilateral PA. It consists in laparoscopic unilateral adrenalectomy.

Although there is no evidence of better outcomes between surgery and optimal medical-treatment, it can be preferred because of benefits regarding blood pressure and potassium concomitant to a reduction in medical treatment and sometimes even hypertension cure and normalization of potassium without medications. This is often the case when patients are young or willing to improve their quality of life by avoiding taking medications for their entire life.

Before proposing AVS and possibility surgery to a patient, it is extremely important to explain that adenoma is not a malignant tumor, that medical treatment, if chosen, is a lifetime treatment, and that neither surgery nor medical treatment is superior in terms of cardiovascular outcomes. If surgery is the final choice and AVS shows unilateral secretion, MRA should nevertheless be prescribed to control HTN before surgery

The outcomes of surgery should also be explained. In patients with lateralized PA, surgery allows normalizing potassium in 95% of patients, curing HTN in 40% of patients, or reducing blood pressure medications in 35 to 60% of other patients [37].

The success after surgery is defined by the PASO criteria (Primary Aldosteronism Surgery Outcome) [38]. They define biological success (complete or partial, according to kalemia and ARR) and clinical success (complete or partial, according to persistence of HTN, with or without treatment).

##### Medical Treatment

Medical treatment for PA is recommended for patients with unlateralized form of PA or for patients who did not undergo AVS. It is based on MRA.

Spironolactone is the first line of treatment. It is a competitive antagonist of aldosterone. Its long half-life can result in delayed efficacy and persistence of side effects such as hyperkaliemia a few days after stopping.

A few years ago, patients were given 1 to 4 mg/kg [39], but as spironolactone is not specific for the mineralocorticoid receptor, side effects related to androgen and progesterone receptors can be inconvenient. These side effects are mostly gynecomastia, breast pain and erectile dysfunction in men and dysmenorrhea in women.

These side effects are dose dependent and are very reduced under 50 mg/day [40]. Moreover, efficacy is unaltered [41]. Therefore, we now use lower dosages.

The second line in the case of intolerance of spironolactone is eplerenone, which is a specific antagonist of mineralocorticoid receptor and is, therefore, free of its side effects. However, the half-life of eplerenone is shorter, and it must be taken twice a day, which affects observance. Its efficacy is also lower [42]. We usually assume that for the same efficacy, dosage must be doubled compared to spironolactone (25 mg of spironolactone equals 25 mg twice a day of eplerenone).

Non-steroidal mineralocorticoid receptor antagonists such as esaxerenone and finerenone are being developed mainly for diabetic nephropathy and heart failure. These drugs might one day be a therapeutic option for the management of PA.

When specific treatments are not sufficient to control HTN, diuretics are very effective. Moreover, a study showed that patients whose renin activity remained suppressed on MRA had a higher risk of CV events [43], which could encourage the use of diuretics in these patients. Diuretics should be prescribed with a strict surveillance of potassium due to the risk of hypokalemia. In the case of persistent hypokalemia, distal diuretics can be used and are very effective (ENaC inhibitors such as amiloride) rather than potassium supplementation.

### 2.2. Other Forms of Mineralocorticoid Excess or Effect

These disorders should be considered in patients presenting hypertension and hypokalemia associated with low concentrations of renin and aldosterone

#### 2.2.1. Congenital Adrenal Hyperplasia

Congenital adrenal hyperplasia is a group of disorders caused by enzymatic defect in adrenal steroidogenesis resulting in a defect in cortisol secretion. In most cases, CAH is a result of 21-hydroxylase deficiency and does not result in hypertension. However, deficiency in 11B hydroxylase and 17 alpha hydroxylase results in an accumulation of 11 desoxycorticosterone (DOC), activating the mineralocorticoid receptor and resulting in hypertension with low renin and aldosterone levels. The diagnosis is confirmed by steroidal profile in mass spectrometry and germline mutation testing (CYP11B1) [44].

#### 2.2.2. Apparent Mineralocorticoid Excess

In the kidney, cortisol, which is a potent mineralocorticoid, is inactivated by a transformation to cortisone by an enzyme: 11 beta hydroxysteroid deshydrogenase type 2. If this enzyme is deficient, high levels of cortisol will be present in the kidney and will bind to mineralocorticoid receptor. Decreased HSD11B2 activity can be a result of a germline mutation in the gene or can be secondary to pharmacologic inhibition by glycyrrhizic acid (licorice) [45].

#### 2.2.3. Liddle Syndrome

Liddle syndrome is an autosomal dominant renal disease secondary to a mutation of the amiloride sensitive epithelial sodium channel. It results in a massive reabsorption of sodium and results in severe hypertension with hypokalemia [46].

#### 2.2.4. Cushing Syndrome

Endogenous hypercortisolism is a rare disease with an incidence of 1 per million people per year. Despite its rareness, it is very important to look for this diagnosis because it results in cardiovascular, infectious, or orthopedic (fractures) complications. The origin is the adrenal glands in 20% of cases, the pituitary gland in 70% of cases, and paraneoplasic in 10% of cases [47].

Typical sign and symptoms include weight gain with central obesity, muscle weakness, fine skin, or purple stretch marks. Hypertensive patients should be screened if they present signs or symptoms consistent with Cushing syndrome. Other manifestations can include hypokalemia, diabetes, dyslipidemia, neutrophilia, or osteoporosis.

Detection usually consists in 1 mg overnight dexamethasone suppression test (cortisol detection at 8 am after administration of 1 mg dexamethasone at midnight). Confirmation tests should include midnight cortisol either in plasma or in saliva and urinary 24 h cortisol excretion. If the diagnosis is confirmed, the next step is the measurement of ACTH at 8 am to precise adrenal or pituitary origin of hypercortisolism and specific imaging (pituitary MRI or adrenal CT, eventually completed with nuclear imaging) [48].

It is highly recommended to check for metabolic and orthopedic complications (fractures and osteoporosis).

Patients with established Cushing syndrome should be oriented to reference centers to discuss treatments (surgery or medical treatment with metyrapone).

#### 2.2.5. Cortisol Resistance

Rare mutations in the glucocorticoid receptor can result in cortisol resistance. These patients do not display clinical signs of hypercortisolism, but they have an accumulation of DOC and cortisol, which can result in hypertension and hypokalemia [49].

#### 2.2.6. Gordon Syndrome

Gordon syndrome is a differential diagnosis of PA and is also known as pseudohypoaldosteronism type II (PHA type II). It is caused by an autosomic dominant mutation in WNK1 and WNK4 genes, which regulate sodium transporter in distal renal tubules. The consequences include severe HTN, hyperkalemia (which is the major difference with the phenotype of PA), and metabolic acidosis. Aldosterone levels are normal or elevated, and renin is low, as in PA [50].

## 3. Pheochromocytoma and Paraganglioma

### 3.1. Definition and Epidemiology

Paragangliomas (PGLs) are rare neuroendocrine tumors arising from sympathetic and parasympathetic ganglia and from the adrenal medulla. Their incidence is estimated between two and eight cases per million per year [51,52].

A pheochromocytoma is a paraganglioma arising from the adrenal medulla.

Among hypertensive patients, the incidence is much higher (between 0.2 and 0.6%), and it reaches 4 to 7% in patients with incidentaloma [48,53,54]. Eighty to eighty-five percent are pheochromocytomas, and 10–15% are sympathetic paragangliomas arising from the sympathetic nervous system in thorax, abdomen, and pelvis. Cervical paragangliomas are developed from the parasympathetic system.

Around 10 to 20% patients develop metastatic lesions sometimes at initial diagnosis, but they are also sometimes discovered during the surveillance [55].

### 3.2. Who Should Be Screened?

Paragangliomas and pheochromocytomas (PPGLs) can be revealed by a compression syndrome or by paroxysmal or permanent signs or symptoms due to an episodic or continuous excessive catecholamine secretion by the tumors. Symptoms are related to the amounts of catecholamines released. Paroxysmal symptoms can be provoked by triggers such as medications, exercise, or surgery [56].

Only functional PPGL with hypersecretion of catecholamine will be treated in this chapter as non-functional PPGLs do not result in hypertension.

Hypertension is present in 80% of patients and a result of increased peripheral resistance, high cardiac frequency, and secondary hyperaldosteronism. This secondary hyperaldosteronism is caused by dehydration due to pressure natriuresis. Patients can also have hypotension or normal blood pressure). Other symptoms include headache (60–90%), palpitations (50–70%), and sweating (55–75%), which define the “Menard Triade,” and, when combined with syncope, have a specificity of 90% for the diagnosis of functional PPGL. Other symptoms include anxiety, hyperglycemia, loss of weight, weakness, or nausea [57].

PPGL can also present with severe cardiovascular complications such as hypertensive emergency, myocardial infarction, or Takotsubo cardiomyopathy.

In brief, screening for PPGL is recommended in the following cases [58]:

1. Signs and symptoms of PPGL: spontaneous or provoked;
2. Cardiovascular events with symptoms of PPGL, Takotsubo cardiomyopathy;
3. Young patients (<50 years) with diabetes type 2 despite BMI < 25 kg/m^2^;
4. Adrenal incidentaloma with density more than 10 HU, even in the absence of hypertension (commonly, all adrenal incidentaloma are screened in our center);
5. Genetic disease or mutation linked to an increased risk of PPGL (see “Genetics”) or family history of PPGL;
6. Even if not recommended by the working group on Endocrine Hypertension of the ESH, screening for PPGL could also be justified in the following cases;
7. High blood pressure variability;
8. Resistant hypertension or sever hypertension (grade 3);
9. Cervical, abdominal or pelvic mass syndrome.

### 3.3. How to Diagnose PPGL

#### 3.3.1. Biochemical Diagnosis

The biological diagnosis consists in highlighting catecholamines hypersecretion.

The best sensibility and specificity are obtained with a dosage of free metanephrine and normetanephrine, which are metabolites of adrenaline and noradrenaline, in blood or 24 h urine [58,59].

The dosage is currently performed by liquid chromatography with electrochemical detection (LC-ECD) or tandem mass spectrometry (LC-MS/MS). LC-MS is preferred because it is more accurate, more cost-effective, and has less drug interactions.

Both plasma and urinary metanephrine and normetanephrine have >99% negative predictive value and around 94% specificity. The diagnostic is almost certain when dosage is >3 N and suspected when dosage is >2 N [60]. Plasma measurements tend to be preferred because they show similar accuracy in patients at low risk for PPGL but higher accuracy in high-risk patients than urine measurements [59]. False positives can be due to activated sympathetic nervous system; thus, the measurements should be performed in stress-free conditions and in supine position [61]. Some medications can interact with the secretion of plasma metanephine and normetanephine (Table 2).

#### 3.3.2. Imaging

After discovery of symptoms and/or catecholamine excess evocating PPGL, imaging is a very important step. It combines anatomic and functional imaging to perform precise topography of the tumors. The choice of imaging modality depends on the type of tumor, its location, and the genotype [62].

**Figure 2:**
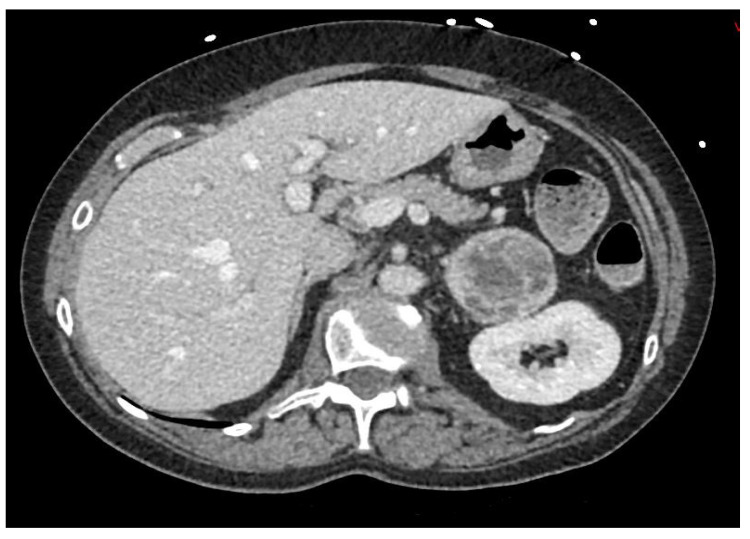
Abdominal CT (DFOV 85 × 37 mm): Pheochromocytoma, left heterogenous adrenal tumor.

Regarding thoracic, abdominal, and pelvic regions, the CT scanner is the first choice. PPGLs have a median size of 5 cm, are hypervascularized, have a necrotic aspect, and a possess a density of more than 10 Hounsfield unit (HU) (Figure 2) [63]. According to two studies, a density of more than 10 HU is found in more than 99.5% of pheochromocytomas, suggesting a great negative predictive value when density is less than 10 HU [64,65].

Functional imaging is recommended for improving sensitivity and specificity, particularly in patients with hereditary syndrome, high risk of multifocality, or metastatic diseases. 18 F DOPA PET or 68 Ga DOTATOC PET are recommended as the first choice [62]. Moreover, DOTATOC is considered the better choice in head and neck PGLs, associated with anatomic imaging with MRI [66]. However, it is not available in every center.

123 I MIBG scintigraphy used to be recommended, but its sensitivity is inferior to CT scan or MRI; moreover, it is very low for PGL. It can still be used for diagnosis confirmation when mass is known and hormonal dosages are not sufficient for conclusion.

### 3.4. Genetics

PPGL are genetically determined in 40% of cases, which is the highest rate among human tumors, and, adding somatic mutations, 80% of PPGL can be explained by a genetic alteration.

They are linked to germline mutations among more than 25 susceptibility genes (SDH A-B-C-D, FH, SLC25A11, MDH2, RET, VHL, NF1, MAX, MET, TMEM127…), and this number increases every year [67,68].

These mutations increase the risk for the patient to develop PPGL. Moreover, some mutations (VHL, NF1, and RET) are associated to other tumors rather than PGL (for instance, RET is associated to multiple endocrine neoplasia, VHL to hemangioblastomas, etc.), while other mutations are associated with metastatic forms of PGL (SDHB, FH, and SLC25A11).

It is, therefore, recommended to propose genetic testing to every patient with PPGL or at least to provide information about the consequences of identifying a genetic form during a genetic consultation. The identification of a genetic form has a positive impact on the follow-up of patients [69]. Moreover, if a genetic form is found, it can allow specific surveillance and screening for first degree relatives. Finally, due to different gene penetrance, the absence of family history does not rule out the presence of a germline mutation.

### 3.5. Treatment

Treatment functional PPGL consists in surgical resection.

The surgery must be performed by a multidisciplinary trained team. Indeed, surgery can result in a massive release of catecholamines in plasma with potential serious cardiac outcomes [70]. There is also a risk of sever hypotension after surgery.

Treatment of hypertension is recommended before surgery. The first choice is alpha-blockers, although this is based on observational studies and expert opinions [58,71]. There is, however, a physio-pathological rationale in blocking alpha-adrenergic receptors to avoid major consequences of catecholamines secretion.

The patients should be hydrated, and alpha blockers should be introduced progressively. Betablockers can only be added once alpha-adrenergic receptors are already blocked in the case of tachycardia in order to avoid paradoxical activation of the sympathetic nervous system.

Aggressive metastatic PPGLs with rapid progression can be treated with chemotherapy or radiotherapy.

### 3.6. Surveillance

Whether the patient had surgery or not, surveillance is always necessary, as patients can develop other PPGLs or metastatic lesions even after complete removal. Indeed, according to WHO 2016, there are no validated histological criteria on the primary lesion for the diagnosis of malignancy of PPGLs; therefore, all have metastatic potential.

Recommendations propose plasma or urinary free metanephrine and normetanephrine measurement 2 to 6 weeks after surgery. In the case of elevated hormones, imaging (CT or MRI) should be performed after 3 to 6 months [72].

Globally, the surveillance in patients with PPGL, whether they had surgery or not, depends on genetics, initial lesions, localization, and metanephrine levels and should be personalized in a discussion with a multidisciplinary expert team [72]. Normally, patients with sporadic PPGL have annual surveillance during at least 10 years (and among them 10 to 15% might present new lesions). Patients with genetic forms should be followed indefinitely [72].

## 4. Other Endocrine Causes of Hypertension

### 4.1. Acromegaly

Acromegaly is defined by increased rate of growth hormone (GH) and insulin-like growth factor 1 (IGF1). Its prevalence in general population ranges from 7 to 13 cases per 100,000 individuals, and hypertension occurs in 20–50% of patients.

Acromegaly should be screened in hypertensive patients (with the measurement of insulin like growth factor 1) when they have clinical manifestations consistent with the diagnosis (a rectangular face, an enlarged, widened nose, prominent cheekbones, thickened lips, prognathism, maxillary, soft tissue overgrowth, and skeletal deformities…) [73].

### 4.2. Thyroid Dysfunction and Primary Hyperparathyroidism

Hyperthyroidism and hypothyroidism have been both associated with hypertension. However, there are no data on the prevalence of hyper and hypothyroidism in patients who present with hypertension. Primary hyperparathyroidism has also been described as a cause of hypertension. Regarding the prevalence of these diseases, it is not known whether thyroid dysfunction and hyperparathyroidism are causes of hypertension or only associated pathologies. The screening for hypo, hyperthyroidism and primary hyperparathyroidism should be performed if patients have clinical signs of hypo/hyperthyroidism or hypercalcemia. Therefore, the indications of screening are the same as in a non-hypertensive population [74].

### 4.3. Secondary Hyperaldosteronism

Renal artery stenosis, renal infarction, rarely renin tumours, and any renal damage can result in a secretion of renin and secondary hyperaldosteronism. Patients with secondary hyperaldosteronism can present with hypertension, which might be associated with hypokalemia. We will not develop these causes, as the primum movens is not endocrine but renal.

## 5. Conclusions

In conclusion, screening for endocrine hypertension should always be performed in young patients and patients with severe hypertension. However, screening should begin with the evaluation of clinical signs and symptoms for the majority of pathologies, except for PPGL and primary aldosteronism. Indeed, clinical signs are rather poor for these pathologies and patients can be asymptomatic. Moreover, PPGL is a life-threatening pathology, and PA is the most common cause of secondary hypertension.

## Figures and Tables

**Table 1:** Effect of medications on plasma renin and aldosterone.

Medication Class	Plasma Aldosterone	Plasma Renin	ARR	Interruption Delay
Diuretics (thiazides or loop)	↑→	↑↑	↓	15 days
ACEi or ARB	↓	↑↑	↓	15 days
Betablockers	↓	↓↓	↑	15 days
NSAI	↓	↓↓	↑	15 days
MRA	↑	↑↑	↓	45 days

→: no effect; ↑: increase; ↓: decrease; ↑↑: significant increase; ↓↓: significant decrease.

**Table 2:** Drug interactions with plasma metanephine and normetanephine.

	Normetanephrine	Metanephrine
Tricyclic antidepressant, phenoxybenzamine	↑↑	↓
MAO inhibitors	↑↑	↑↑
Bétablockers	↑	↑
Alphamethyldopa, levodopa	↓	↓

→: no effect; ↑: increase; ↓: decrease; ↑↑: significant increase; ↓↓: significant decrease.

## Data Availability

Not applicable.

## Abbreviations

ACEi	angiotensin-converting enzyme inhibitors
APA	aldosterone-producing adenomas
ARB	angiotensin2 receptor blockers
ARR	aldosterone-to-renin ratio
AVS	adrenal vein sampling
CCB	calcium channel blockers
CT	computed tomography
CV	cardiovascular
DALYs	disability-adjusted-life-years
DBP	diastolic blood pressure
DOC	desoxycorticosterone
DRC	direct renin concentration
GRA	Glucocorticoid remediable aldosteronism
MRA	mineralocorticoid receptor antagonists
MRI	magnetic resonance imaging
HTN	hypertension
PA	primary aldosteronism
PCR	polymerase chain reaction
PHA	pseudohypoaldosteronism
PGL	Paragangliomas
PPGL	pheochromocytoma and paraganglioma
PRA	plasma renin activity
RAS	renin-angiotensin system
RASb	renin-angiotensin system blockers
